# Maresin 1 induces resolution of hepatic fibrosis through RORα-dependent reprogramming of resident macrophages

**DOI:** 10.3389/fphar.2026.1826739

**Published:** 2026-04-27

**Authors:** Francisca Herrera Vielma, Matías Quiñones San Martin, Nicolás Muñoz-Carrasco, Fernanda Berrocal-Navarrete, Andrés A. Herrada, Alexandra Olate-Briones, Sebastián Zagmutt, Rosalía Rodríguez-Rodríguez, Daniel R. González, Jessica Zúñiga-Hernandez

**Affiliations:** 1 Pharmacology Laboratory, Biomedical Sciences Department, Faculty of Health Sciences, University of Talca, Talca, Chile; 2 Institute of Chemistry of Natural Resources, University of Talca, Talca, Chile; 3 School of Biotechnology Engineering, Faculty of Agricultural and Forestry Sciences, Catholic University of Maule, Talca, Chile; 4 Faculty of Health Sciences, University of Talca, Talca, Chile; 5 Lymphatic Vasculature and Inflammation Research Laboratory, Department of Clinical Biochemistry and Immunology, Pharmacy Faculty, Universidad de Concepción, Concepción, Chile; 6 Basic Sciences Department, Faculty of Medicine and Health Sciences, Universitat Internacional de Catalunya, Sant Cugat del Vallès, Spain

**Keywords:** hepatic fibrosis, macrophage polarization, Maresin 1, NF-κB, pro-resolving mediators, RORα

## Abstract

Hepatic fibrosis is a progressive pathological condition characterized by chronic inflammation and excessive extracellular matrix deposition (ECM), which may progress to cirrhosis and liver failure. Although specialized pro-resolving lipid mediators have emerged as potential therapeutic agents, their role in advanced hepatic fibrosis remains incompletely defined. This study aimed to determine whether Maresin 1 (MaR1), an endogenous pro-resolving lipid mediator, promotes the resolution of hepatic fibrosis and modulates the associated inflammatory response. Hepatic fibrosis was induced in mice and rats using diethylnitrosamine (DEN). Animals were subsequently treated with MaR1 or vehicle. Histological and biochemical parameters, apoptosis and proliferation markers, and immune profiles associated with hepatic macrophages polarization were assessed. Additionally, the expression and subcellular localization of retinoic acid related orphan receptor alpha (RORα) and nuclear factor kappa B (NF-κB p65) were analysed. MaR1 treatment significantly attenuated hepatic fibrosis, reduced the ECM accumulation, and promoted restoration of liver parenchyma, accompanied by decreased hepatocellular injury and enhanced regenerative capacity. MaR1 also induced immune reprogramming, favouring anti-inflammatory and homeostatic macrophage phenotypes. These effects were associated with increased nuclear activation of RORα and modulation of NF-κB signalling pathways. These findings demonstrate that MaR1 promotes the resolution of hepatic fibrosis through macrophage polarization and modulation of the RORα/NF-κB axis. This study advances the understanding of pro-resolving mechanisms in hepatic fibrosis and positions MaR1 as a pharmacologically relevant candidate for the development of targeted antifibrotic therapies in chronic liver disease.

## Graphical Abstract

**Figure:**
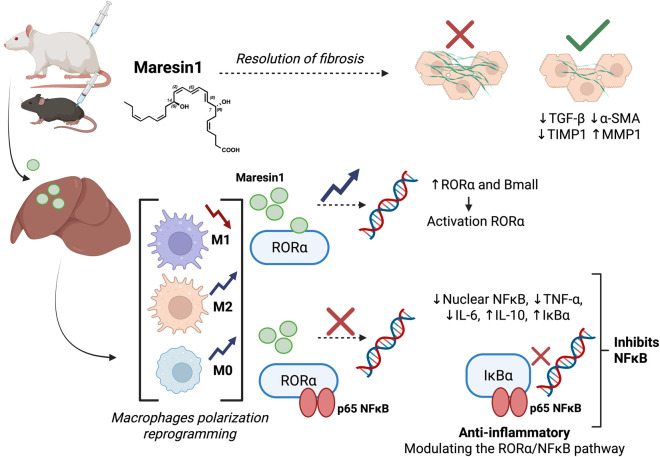

## 1 Introduction

Fibrosis is a central pathological feature of chronic liver diseases and represents a major determinant of progression toward cirrhosis and liver failure (Friedman, 2008). It is characterized by an excessive scarring response that develops following sustained hepatic injury and chronic inflammation, leading to the accumulation of extracellular matrix (ECM) and distortion of liver architecture (Friedman, 2003). This process results from an imbalance between ECM synthesis and degradation in response to repetitive or persistent insults of sufficient intensity, ultimately compromising hepatocellular function and tissue homeostasis (Altamirano-Barrera et al., 2017).

Macrophages are pivotal regulators of liver injury, fibrogenesis, and tissue repair (Wynn and Vannella, 2016). In the hepatic microenvironment, two main macrophage populations coexist: resident Kupffer cells (KCs) and monocytes-derived macrophages (MoMas), which are recruited from the circulation in response to liver injury. These cells exhibit marked phenotypic plasticity and can adopt a proinflammatory phenotype (M1), characterized by the production of cytokines such as tumour necrosis factor-alpha (TNF-α) and interleukin-1 beta (IL-1β) (Mosser and Edwards, 2008), thereby exacerbating tissue damage and fibrogenesis. Conversely, macrophages can acquire an anti-inflammatory (M2) phenotype, associated with the secretion of IL-10 and other mediators that promote ECM remodelling and tissue regeneration. The dynamic balance between these macrophage activation states is therefore critical for determining of whether liver injury progresses toward fibrosis or undergoes resolution (Krenkel and Tacke, 2017).

Lipid derived mediators have emerged as key regulators of hepatic inflammation and macrophage behaviour. In this context, docosahexaenoic acid (DHA) and its bioactive derivates have been shown to attenuate liver injury by promoting the biosynthesis of specialized pro-resolving mediators (SPMs) (Videla et al., 2023). Among these, maresins (MaRs) constitute a family of endogenous lipid mediators with potent anti-inflammatory and pro-resolving actions. MaR1 has demonstrated beneficial effects in metabolic disorders, including obesity, insulin resistance, and hepatic steatosis (Laiglesia et al., 2018; Martínez-Fernández et al., 2017), and has been shown to drive macrophage polarization toward reparative phenotypes (Bi et al., 2019; Qiao et al., 2020; Zhu et al., 2016).

In parallel, nuclear receptors play a central role in integrating metabolic and inflammatory signalling pathways that govern liver homeostasis (Weikum et al., 2018). RORα has emerged as a key regulator of lipid metabolism, oxidative stress, and inflammatory responses in the liver (Jetten, 2009). Clinical and experimental evidence indicates that RORα expression is reduced in metabolic dysfunction-associated steatotic liver disease (MAFLD) and dysfunction-associated steatohepatitis (MASH) (Han et al., 2014; Ou et al., 2012). In addition, peroxisome proliferator-activated receptor alpha (PPARα) is a well-established regulator of hepatic fatty acid β-oxidation and inflammatory control (Pawlak et al., 2015), underscoring the importance of the nuclear receptor network in shaping the hepatic immunometabolic environment (Jamieson et al., 2017). Notably, recent evidence has identified RORα as a functional receptor for MaR1, mediating hepatoprotective effects through transcriptional regulation of genes involved in lipid metabolism, oxidative stress responses, mitochondrial function, and macrophage polarization (Han et al., 2014). Despite these advances, the mechanism by which MaR1-driven activation of RORα integrates inflammatory signalling and macrophage reprogramming during liver fibrosis remains incompletely understood. In the present study, we hypothesized that MaR1 promotes hepatic fibrosis resolution by activating RORα, reprogramming macrophage responses and suppressing NF-κB–dependent inflammation. To test this hypothesis, we examined the effects of MaR1 on hepatic injury, macrophage polarization, and nuclear receptor signalling in murine models of liver fibrosis, with particular emphasis on the RORα–NF-κB axis. Our findings identify a mechanistic link between pro-resolving lipid mediator signalling and nuclear receptor mediated control of hepatic inflammation, positioning MaR1 as a promising pharmacological candidate for the treatment of chronic inflammatory and metabolic liver disease.

## 2 Materials and methods

### 2.1 Animal preparation

Youth male Sprague-Dawley (SD) rats (70–90 g, approximately 3–4 weeks old) and C57BL/6 mice (19–20 g, approximately 5–6 weeks old) were obtained from the Central animal facility of the University of Talca. Animals were housed under controlled temperature and a 15-h light/dark cycle with food (Prolab® IsoPro® RMH 3000, United States) and water *ad libitum*. They were randomly assigned to four experimental groups (n = 6 per group): control group (vehicle + vehicle), DEN group (DEN + vehicle); MaR1 group (vehicle + MaR1), and MaR1/DEN group (DEN + MaR1). Sample sizes were determined based on previous studies from our group and comparable experimental models of liver fibrosis to ensure adequate statistical power.

Liver fibrosis was induced by intraperitoneal (i.p.) administration of DEN (Cat No. 73861, Sigma-Aldrich, Merck, Darmstadt, Germany) at a dose of 50 mg/kg body weight, two times a week for a period of 4 weeks and then once weekly for an additional 5 weeks, according to the DEN-induced hepatic fibrosis model (Guo et al., 2019). C57BL/6 mice received DEN at 25 mg/kg following the same administration schedule (Kushida et al., 2011).

MaR1 (Cat. No. 10878, Cayman Chemical, Ann Arbor, MI, United States) was administered once daily (4 ng/g of body weight) from week 5–9, based on doses previously validated in our laboratory (Rodríguez et al., 2021). At week 9, animals were fasted (6–8 h) and anesthetized with acepromazine maleate, xylazine, and ketamine hydrochloride (Supplementary Table S1).

### 2.2 Pharmacokinetic study and tissue distribution analysis

#### 2.2.1 Pharmacokinetics study

Four SD rats (350–450 g) were used per time point. Animals received a single i.p. dose of MaR1 (4 ng/g body weight). Plasma and tissues were obtained at 0, 0.5, 1, 3 and 6 h after MaR1 administration. Pharmacokinetic parameters (Tmax, Cmax, AUC_0_–t, AUC_0_– ∞ , CL obs, Kel, and t_1_/_2_) were calculated by non-compartmental analysis using PKSolver 2.0 (Microsoft Excel add-in).

#### 2.2.2 Tissue distribution study

Samples collected in the pharmacokinetic study were used to assess MaR1 and its derivatives at 0 and Tmax in plasma, liver, kidney, and heart. Quantification was performed in duplicate using a commercial ELISA kit (Maresin 1, cat. no. 501150, Cayman Chemical, United States), according to the manufacturer’s intructions.

### 2.3 Determination of biochemical parameters

Serum alanine aminotransferase (ALT), aspartate aminotransferase (AST), albumin and bilirubin were measured with commercial diagnostic kits (ALT, AST Valtke ® Diagnostic Kits, Ñuñoa, Chile; Albumin Human™, Wiesbaden, Germany, Bilirubin Human™, Wiesbaden, Germany). Normal and pathological controls were included. These analyses confirmed biochemical alteration associated with liver injury and the hepatoprotective effect of MaR1 observed in our previous study (Rodríguez et al., 2021).

### 2.4 Liver histology

Liver samples were fixed in 10% phosphate-buffered formalin, processed using a Leica TP 1020TM automatic tissue processor, and embedded in paraffin using a Leica, EG11504TM system. Sections (5 µm) were obtained and stained with haematoxylin and eosin (H&E) and Masson’s trichrome to evaluate morphology and fibrosis. Staining reagents, including Weigert’s Haematoxylin for Masson’s contrast, were purchased from Merck. Histological scoring was performed in duplicate on five random fields per section using the Korourian score (Korourian et al., 1999). In addition, the mitotic index was determined by counting the number of mitotic cells per field in five randomly selected fields per slide. Cells were identified as mitotic when hepatocytes exhibited clearly distinguishable mitotic figures, particularly anaphase and telophase, to ensure accurate identification, while avoiding the inclusion of apoptotic and necrotic cells. The counting cells were observed at ×400 magnification. Collagen type I deposition was quantified as the percentage of stained areas in Masson’s trichrome-stained sections. Analyses were conducted Leica DM500 (Leica Microsystems, Heerbrugg, Switzerland), and image quantification was performed using ImageJ.

### 2.5 Immunohistochemistry

After deparaffinization and hydration, antigen retrieval was performed at 95 °C in citrate buffer (10 mM sodium citrate, 0.05% Tween 20, pH 6.0). Endogenous peroxidase activity was quenched with 3% H_2_O_2_, and non-specific binding was blocked in PBS, containing 0.5% bovine serum albumin, 0.3% triton X-100). Liver sections were incubated with collagen type I (anti-COL1A1 monoclonal antibody, clone 3G3, Cat. No. sc-293182, Santa Cruz Biotechnology), or anti-Ki67 (monoclonal 1:500, Cat. No. sc-23900, Santa Cruz Biotechnology). For Ki67 analysis, immunostaining was considered positive only in cells exhibiting clear and well-defined nuclear. Membranous or matrix signals were not included in the quantification and were interpreted as non-specific background staining. The ABC kit (Vector Laboratories Inc., Burlingame, CA, United States) was used for biotinylated secondary antibody, and samples were developed using the ImmPACT® DAB kit (Vector Laboratories Inc.). Harris haematoxylin was used as a counterstain. The percentage of Ki67-positive nuclei was quantified using ImageJ software, as described above.

### 2.6 TUNEL assay

Apoptosis was evaluated by detecting DNA fragmentation using the TUNEL DeadEnd™ fluorometric system (Promega, Madison, WI, United States), following the manufacturer´s instructions.

### 2.7 Flow cytometry

Mice were anesthetized, the livers were surgically removed, and reperfused (PBS 1X). Then, livers were minced and enzymatically digested (Liberase™ (Sigma Aldrich), centrifuged and filtered. The cells obtained were incubated with fluorescent-conjugated antibodies in PBS, containing 2% (w/v) bovine serum albumin. The following fluorescently labelled antibodies and dyes were used: 7-AAD-PerCP (Cat No. 420403, Bio Legend), CD31-FITC, F4/80-PE (clone BM8.1, Sigma Aldrich), CD11b-PerCP (clone M1/70, Bio Legend), CD206-APC (clone C068C2, Bio Legend), CD11c-PeCy7 (clone N418, Bio Legend), CD45-APC-Cy7 (clone 30-F11, Bio Legend), and CD86-Pacific Blue (clone GL1, Bio Legend). Samples were analysed by flow cytometry using a BD FACSCanto II instrument (BD Bioscience, San Jose, CA, United States) and data were analysed using FlowJo version X.0.7 (Tree Star, Inc., Ashland, OR, United States). The gating strategy used to identify the different cell populations, as well as additional controls, are shown in Supplementary Figure S1.

### 2.8 Immunofluorescence for macrophage phenotype analysis

After deparaffinization and antigen retrieval (95 °C, 10 mM citrate buffer, 0.05% Tween 20 pH 6.0), liver sections were incubated with mouse monoclonal anti CD86 (1:200, Santa Cruz Biotechnology), or anti CD163 (1:200, Santa Cruz Biotechnology) followed by FITC or TRITC-conjugated secondary antibodies (Jackson Immunoresearch, West Grove, PA, United States). Fluorescence was analysed using a Zeiss microscope with Zen software (Carl Zeiss, Jena, Germany). Five random fields per section were evaluated.

### 2.9 Immunofluorescence for nuclear receptors

Liver samples were perfused with PBS, fixed in 10% phosphate buffered formalin, cryoprotected in 30% sucrose, and embedded in OCT compound (Tissue-Tek, Cat#4583). Cryosections (30 µm) were permeabilized, blocked, and incubated overnight at 4 °C with primary antibodies against RORα (1:100, Proteintech) and anti NFκB (1:100, Cell Signalling Technology). Sections were then incubated with Alexa Fluor™ 488 or 568 conjugated secondary antibodies (1:1000, Invitrogen) and counterstained with Hoechst 33342 Ready Flow™ dye. Images were acquired using a LEICA TCS SP8 MP multiphoton microscope and analysed with ImageJ software.

### 2.10 Western blotting

Protein samples were separated by SDS-PAGE and transferred to nitrocellulose membranes, Membranes were blocked in TBS-T containing 5% milk or bovine serum albumin, and incubated with primary antibodies, as detailed in Supplementary Table S2, followed by HRP-conjugated anti-mouse (Cell Signalling No. 7074S) or anti-rabbit (Cell Signalling No. 7076S) secondary antibody. Signals were detected using the Omega Lum ® System (Aplegen, San Francisco, CA, United States) and quantified with ImageJ software.

### 2.11 Quantitative real-time PCR

Total RNA was extracted from rat liver tissue using TRIzol™ LS reagent (Invitrogen) and reverse transcribed into cDNA with the PrimeScript kit (Takara, Osaka, Japan). qPCR was performed on Rapid Real-Time PCR (Agilent) and CFX Opus 96 (Bio-Rad) systems using SYBR Green. Gene expression was normalized to HPRT or GAPDH. Primer sequences are listed in Supplementary Table S3.

### 2.12 Statistical analysis

All data are presented as means ± SD. The number of samples is indicated in each figure. Statistical analyses were performed using GraphPad Prism® version 8.0 software (GraphPad Software, Inc. San Diego, CA, United States). “Analysis of variance (one-way ANOVA) was performed after confirmation of normal distribution. As a post-hoc test, Tukey’s multiple comparison test. The value of p < 0.05 was considered significant. All technical replicates were included in the graphs and statistical analyses, and results are expressed as mean ± SD.

## 3 Results

### 3.1 Pharmacokinetics and tissue distribution of MaR1

**FIGURE 1:**
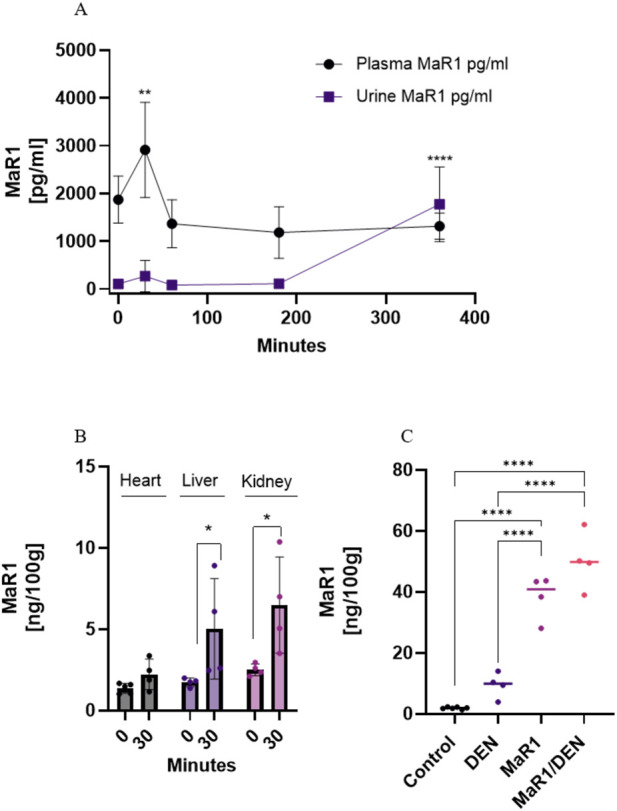
Pharmacokinetics and tissue distribution of MaR1 in SD rats. **(A)** Concentration-time curve of MaR1 in plasma after a single dose of 4 ng/g MaR1 i.p. **(B)** Biodistribution of MaR1 in liver, kidney, and heart tissue at 0 (T0) and 30 min (T0.5) after administration. **(C)** Bioavailability of MaR1 in liver tissue at the end of the experiment (5 weeks) in control, (DEN)-treated, MaR1-treated and MaR1/DEN treated rats. n = 4 animals per group. The asterisk indicates *p < 0.05, **** p < 0.0001.

First, we aimed to characterize the pharmacokinetics of MaR1 administration. Following a single intraperitoneal dose of MaR1 (4 ng/g), plasma levels peaked at 30 min and declined thereafter, reaching low levels by 6 h, consistent with rapid systemic clearance, a characteristic profile of specialized pro-resolving lipid mediators (Figure 1A; Supplementary Table S4). MaR1 rapidly distributed to the liver, kidney, and heart, showing 2-3-fold increases compared with baseline at 30 min (Figure 1B). After chronic administration (35 days), MaR1 accumulated in the liver, particularly in the MaR1/DEN group, which exhibited the highest tissue levels (Figure 1C).

### 3.2 MaR1 decreases parameters of chronic liver injury

To evaluate the impact of MaR1 on chronic liver injury, biochemical and histological parameters indicative of hepatic dysfunction were assessed. DEN administration increased plasma AST, ALT, and bilirubin, while reducing albumin, indicating hepatic dysfunction (Supplementary Figures S2A–D). Histological analyses confirmed extensive fibrosis, necrosis, and inflammatory cell infiltration (Supplementary Figures S2E–H). MaR1 treatment significantly attenuated these alterations, demonstrating a marked hepatoprotective effect in the context of chronic liver injury.

### 3.3 MaR1 exerts an antifibrogenic response

**FIGURE 2:**
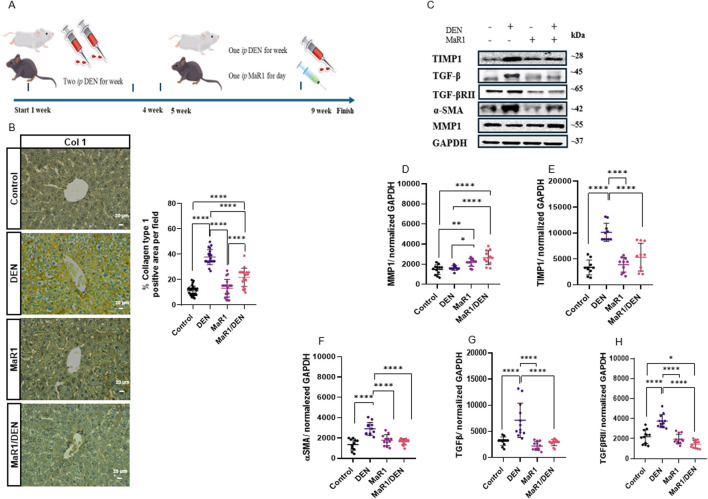
Effect of treatment with MaR1 on extracellular matrix deposition in SD rats with chronic liver injury. **(A)** Experimental design of the murine model showing the induction of chronic liver injury with DEN and treatment with MaR1. **(B)** Representative micrographs of immunohistochemistry for type I collagen in liver sections and quantification of the percentage of positive areas. At least 20 fields of each sample were analysed at ×400 magnification. The scale bar indicates 20 μm. n = 6 animals per experimental group. **(C–H)** Panel of representative Western Blots for MMP1, TIMP1, α-SMA, TGF-β, TGF-β RII, and their quantification, normalized to GAPDH. n = 6 per experimental group. The asterisk indicates *p < 0.05, ** p < 0.005, **** p < 0.0001.

In a murine model of DEN-induced chronic liver injury (Figure 2A), MaR1 significantly attenuated hepatic fibrosis. Immunostaining for type I collagen and histological analysis confirmed increased collagen deposition in DEN-treated mice, which was markedly reduced by MaR1 co-treatment (Figure 2B; Supplementary Figure S3A). DEN- induced upregulation of fibrosis-associated proteins, including inhibitor of metalloproteinases 1 (TIMP1), alpha-smooth muscle actin (α-SMA), transforming growth factor beta (TGF-β), TGF-βRII and concomitant downregulation of matrix metalloproteinase 1 (MMP1), were reversed by MaR1 (Figures 2C–H). MaR1 alone did not affect liver morphology or fibrosis related proteins. These results highlight the antifibrogenic effects of MaR1 at both histological and molecular levels. Notably, the coordinated reduction of profibrogenic markers and restoration of MMP1 expression support and active role of MaR1 in extracellular matrix remodelling rather than merely preventing fibrotic progression.

### 3.4 MaR1 stimulates hepatocellular regeneration in the context of liver fibrosis

**FIGURE 3:**
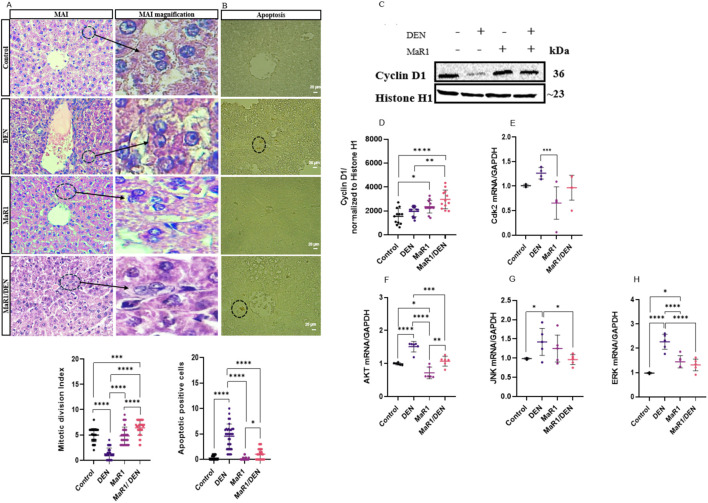
Impact of the MaR1 treatment on proliferation and apoptosis in Sprague- Dawley rats with chronic liver injury. **(A)** Representative H&E sections showing the mitotic activity index (MAI) (left panel) and corresponding magnified images (MAI magnification, middle panel), The corresponding quantification is shown below the columns. **(B)** Representative TUNEL photomicrographs showing apoptotic cells, with corresponding quantification (right panel). The scale bar indicates 20 μm. n = 6 animals per experimental group. Black arrows indicate dividing cells and apoptotic cells. **(C,D)** Representative Western blots for cyclin D1 from liver extracts and quantification, normalized to histone H1. n = 6 per experimental group. **(E)** mRNA levels of Cdk2 **(F)** mRNA levels of Akt **(G)** mRNA levels of JNK **(H)** mRNA levels of ERK. n = 4 per experimental group. The asterisks indicate p < 0.05, * p < 0.005, * p < 0.0005, ** p < 0.0001.

To evaluate the proliferative response to MaR1 in hepatocytes, Ki67, mitotic activity index, and cyclin D1 levels were assessed (Figures 3A–C; Supplementary Figure S4A). Ki67 expression was analysed to corroborate the immunohistochemical findings and to complement the evaluation of cyclin D1, given the potential involvement of kinase-mediated signalling pathways. MaR1 markedly increased hepatocyte proliferation and cyclin D1 expression while reducing apoptosis to levels comparable to controls. The MaR1 group alone showed no significant differences compared to controls. Analysis of kinases signalling pathways revealed that MaR1/DEN reduced Cyclin-Dependent Kinase 2 (Cdk2) mRNA levels, decreased Akt, and attenuated pro-apoptotic kinases c-Jun N-terminal kinase (JNK) and extracellular signal-regulated kinase (ERK), reaching values similar to those observed in control animals. Altogether, these data indicate that MaR1 attenuates pro-apoptotic kinase signalling while promoting hepatocellular proliferation and regeneration. Importantly, this proliferative response occurred in the setting of reduced fibrotic burden and apoptotic signalling, supporting a regenerative rather than pathological hepatocellular proliferation.

### 3.5 MaR1 reprograms hepatic macrophages toward a pro-resolving phenotype

**FIGURE 4:**
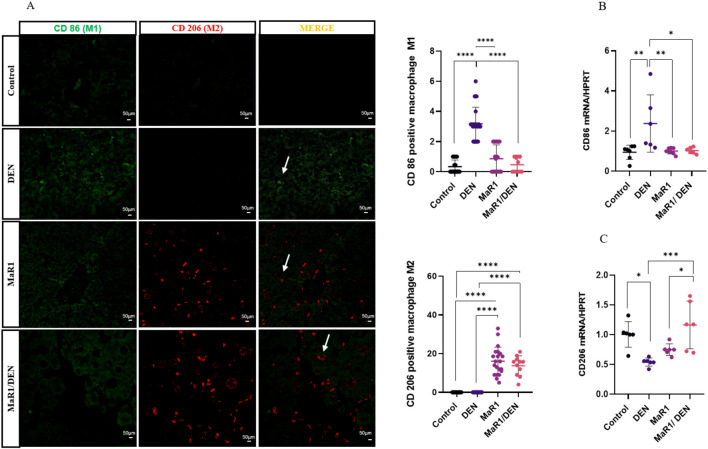
MaR1 reduced M1 macrophages and increased M2 macrophages in hepatic fibrosis in SD rats. **(A)** Representative immunofluorescence images of liver sections from control, DEN-treated, MaR1-treated and MaR1/DEN treated rats, stained with anti-CD 86 (red) and anti-CD 206 (green), and quantification of the number of M1 and M2 cells per field. White arrows show an example of M1 and M2 macrophages. n = 3 rats per experimental group, image fields measured per rat >5. Scale bar indicates 50 µm. **(B)** mRNA levels of CD86 and **(C)** mRNA levels of CD 206, analysed by qRT-PCR. n = 4 per experimental group. The asterisk indicates *p < 0.05, ** p < 0.005, *** p < 0.0005, **** p < 0.0001. CD86: Cluster of differentiation 86; CD206: cluster of differentiation 206/mannose receptor C-type 1; HPRT: hypoxanthine guanine phosphoribosyltransferase.

**FIGURE 5:**
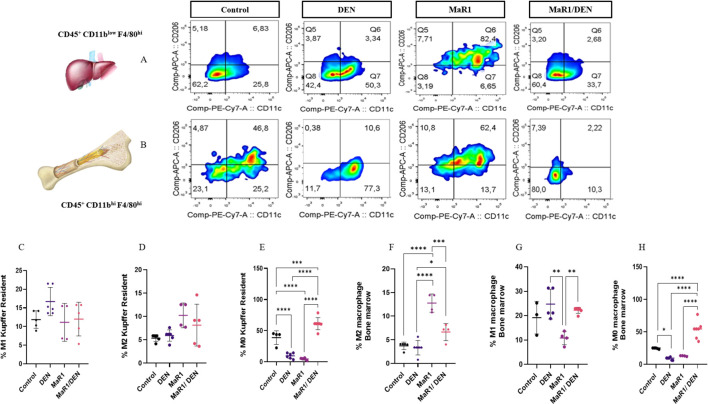
MaR1 induces the polarization of macrophages from a proinflammatory to an anti-inflammatory phenotype in c57bl/6 mice. Flow-cytometry analysis of control, DEN-treated, MaR1-treated and MaR1 + DEN treated C57BL/6 mice for: **(A)** Identification of M1 and M2 macrophages by surface markers CD11c and CD206 from F4/80^hi^ CD45^+^ CD11b^low^ (CKs) using density graphs. **(B)** Identification of M1 and M2 macrophages by surface markers CD11c and CD206 from F4/80^hi^ CD45^+^ CD11b^hi^ (MoMas) using density plots. **(C)** M1 percentage of CKs. **(D)** Percentage of M2 CKs. **(E)** Percentage of M0 cells in CKs. **(F)** Percentage of M2 MoMas. **(G)** Percentage of M1 MoMas. **(H)** Percentage of M0 cells in MoMas. The asterisk * indicates p < 0.05, ** p < 0.005, *** p < 0.0005, **** p < 0.0001. Data from n = 5 mice per group.

Macrophages play a vital role in liver inflammation and repair. Therefore, we evaluated the impact of the MaR1 treatment on their immune profile in fibrosis (Figures 4, 5). Immunofluorescence revealed that the DEN treatment increased pro-inflammatory M1-like macrophages, whereas the MaR1/DEN group exhibited s reduction in M1 and marked increase in anti-inflammatory M2 macrophages. These findings were supported by gene expression of CD86 and CD206 (Figures 4B,C). Using flow cytometry, we distinguished resident KCs (Figures 5C–E) and MoMas, (Figures 5F–H). In both populations, MaR1 decreased M1, increased M2 and promoted expansion of M0 macrophages, indicating a shift toward a homeostatic and pro-resolving macrophage phenotype. MaR1 alone modestly increased M2 macrophages in MoMas, without affecting KCs. Together, these findings strongly suggest that MaR1 acts as an immunomodulatory agent that reprograms both resident and infiltrating hepatic macrophages toward a homeostatic and pro-resolving phenotype.

### 3.6 Modulation of the RORα/NF-κB axis by MaR1

**FIGURE 6:**
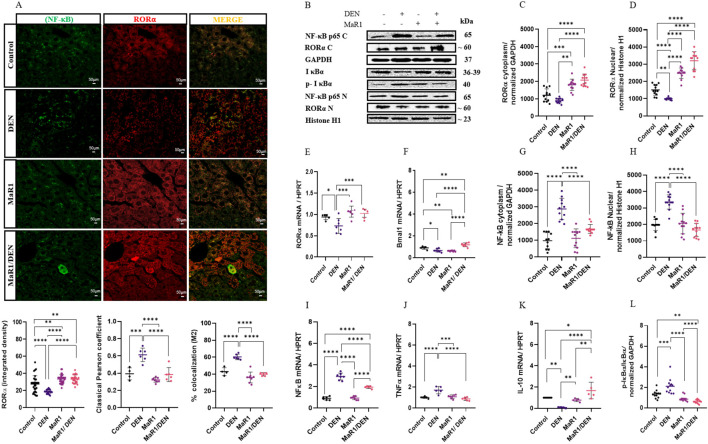
MaR1 modulates inflammation in liver fibrosis through colocalization between RORα and NF-κB in c57bl/6 mice. **(A)** Quantification of RORα expression by immunofluorescence in control, DEN-treated, MaR1-treated and MaR1/DEN treated C57BL/6 mice. Representative IF micrographs of RORα-positive (red) and cytoplasmic NF-κB p65 (green) liver tissue and overlaid quantification of RORα and NF-κB p65 colocalization (yellow). Pearson’s correlation coefficient and Manders’ coefficient (M2) are shown. At least 20 fields of each sample were analysed at ×400 magnification. Scale bar indicates 50 μm. n = 4 per experimental group. **(B)** Representative Western blot images. **(C)** Quantification of RORα protein in cytoplasmic fractions. **(D)** Quantification of RORα protein in nuclear fractions. **(E)** Relative mRNA expression of RORα by RT-qPCR. **(F)** Relative mRNA expression of the RORα target gene BMAL1 by RT-qPCR. **(G)** NF-κB p65 in cytoplasmic fractions with quantification. **(H)** NF-κB p65 in nuclear fractions with quantification. **(I)** Relative mRNA expression of NF-κB by RT-qPCR. **(J)** Relative mRNA expression of TNF-α by RT-qPCR. **(K)** Relative mRNA expression of IL-10 by RT-qPCR. **(L)** Quantification of IκBα and p-IκBα. Western blot levels were normalized to GAPDH, and nuclear levels to histone H1 (n = 6 per experimental group). RT-qPCR values were normalized to HPRT (n = 4 per experimental group). The asterisk indicates *p < 0.05, ** p < 0.005, *** p < 0.0005, **** p < 0.0001. BMAL1: brain and muscle ARNT-like 1; IκBα: inhibitor of kappa B alpha.

Next, we evaluated the impact of the MaR1 treatment in the RORα-NF-κB signalling axis, give that RORα is a receptor for MaR1 in macrophages. In the animals with DEN-induced liver fibrosis, RORα expression was significantly reduced, whereas in the group MaR1/DEN, RORα levels were in both cytoplasmic and nuclear fractions (Figures 6A–D). MaR1 alone increased RORα expression compared with control animals. Colocalization analysis showed that DEN exposure increased the spatial proximity between RORα and NF-κB p65, whereas MaR1/DEN treatment markedly reduced this colocalization, consistent with attenuation of NF-κB signalling. At the transcriptional level, MaR1/DEN restored the expression of RORα and its downstream target gene: brain and muscle ARNT (Bmal1), both of which were decreased by DEN (Figures 6E,F). DEN induced NF-κB p65 activation and nuclear translocation, accompanied by increased phosphorylation of IκBα (p-IκBα). In contrast, MaR1/DEN treatment was associated with reduced NF-κB p65 activation, normalization of TNFα and anti- IL-10 expression, pro-inflammatory and inflammatory cytokines, respectively (Figures 6G–K). Consistently, MaR1/DEN reduced IκBα phosphorylation (Figure 6L). Together, these data suggest that MaR1 pharmacologically restores RORα activity, resulting in suppression of NF-κB signalling and downstream inflammatory gene expression. Importantly, these findings identifies the MaR1/RORα axis as a central regulatory node linking macrophage reprogramming to NF-κB dependent inflammatory pathways during liver fibrosis.

## 4 Discussion

Hepatic fibrosis is a pathology that, if not treated timely, can evolve into cirrhosis and liver failure (Domínguez et al., 2009). Historically, liver fibrosis has been considered irreversible. However, current reports indicate that this phenomenon may be reversible (Bataller and Brenner, 2005). This regression involves a series of mechanisms such as modulation of the inflammatory microenvironment, degradation of the ECM, reduction of oxidative stress and regeneration of hepatocytes (Arthur, 2001; Caligiuri et al., 2021; Kisseleva and Brenner, 2020). In fact, the only effective treatment to attenuate or reverse liver fibrosis is the elimination of the causative agent (Altamirano-Barrera et al., 2017). However, this approach presents several limitations, especially in advanced stages of the disease or in conditions where the etiologic agent cannot be completely controlled. Interestingly, studies have shown that DHA and its derivative MaR1 induce important antifibrotic effects in experimental models of liver disease (He et al., 2017; Rodríguez et al., 2021).

Accordingly, we have focused on MaR1 pharmacokinetic behaviour and its tissue distribution. Our data reveals rapid absorption after the *i.p*. administration, followed by systemic distribution, with peak plasma concentration after 30 min of the injection. This profile was accompanied by a progressive increase in urinary levels, suggesting predominant renal elimination, through glomerular filtration. Importantly, sustained hepatic accumulation of MaR1 was observed following chronic administration, particularly in the MaR1/DEN group at the end of the protocol, suggesting not only relevant bioavailability but also potential tissue retention under conditions of chronic inflammation. Interestingly, DEN-induced inflammation also increased hepatic MaR1 levels in the absence of exogenous treatment, which may result from enhanced release from membrane phospholipids in response to injury. These findings are consistent with previous studies showing MaR1 accumulation in the liver in acute models, such as ischemia/reperfusion (I/R) (Tang et al., 2021), and in chronic fibrosis models, where MaR1 modulated Nrf2 and NF-κB pathways (Rodríguez et al., 2021). Recently, it has been highlighted the role of MaR1 in MAFLD pathophysiology (Videla et al., 2023), and clinical studies have reported reduced serum levels in patients with MAFLD patients (Tang et al., 2021). Moreover, the levels of MaR1 found in the kidney and heart support its potential as a multi-organ therapeutic agent, as indicated by previous evidence of its protective effects in acute kidney injury (*via* NOX4/ROS/NF-κB p65) (Li et al., 2021) and myocardial infarction (*via* Nrf2/HO-1 and TLR4/NF-κB) (Wang et al., 2022). Altogether, these results provide compelling evidence that MaR1 exhibits favourable bioavailability, reaches key organs affected by chronic inflammation, and may modulate pathophysiological responses beyond the liver.

Regarding fibrosis, as in our previous report, MaR1 reversed the biochemical and histopathological parameters once liver fibrosis was established, reducing the excessive deposition of ECM. Furthermore, it facilitated the entry of hepatocytes into the cell cycle, enhancing proliferation, and reducing apoptosis (Rodríguez et al., 2021). In parallel, an innovative aspect of our study was the identification of MAPKs as participants in the process of liver fibrosis. Although this mechanism has not been widely explored in liver fibrosis, previous studies have showed that MaR1 modulates MAPK pathways in different tissue injury models, such as renal injury and sepsis, suggesting a protective role mediated through the inhibition of these signalling pathways (Dai et al., 2025; Sun et al., 2019). In our experiments, we observed that JNK expression decreased after MaR1 administration. JNK is activated in response to stress signals, such as stimulation or production of proinflammatory cytokines, including TNF-α, following activation of KCs (Lentsch et al., 1999; Uehara et al., 2005). In the case of the ERK pathway, it promoted liver apoptosis and contributed to fibrosis. Recent studies have explored therapeutic strategies directed at receptors that modulate this pathway reducing the development of liver fibrosis (Zhang et al., 2024). Regarding cell survival and proliferation, a decrease in Akt signalling was observed following MaR1 administration in liver fibrosis. In this context, modulation of the Akt pathway may be beneficial, as sustained or dysregulated Akt activation has been associated with enhanced survival and proliferation of HSCs, thereby promoting ECM accumulation and accelerating fibrosis progression (Shamsan et al., 2024). Thus, the reduction of Akt signalling observed here may contribute to the attenuation of fibrogenic responses. Previous studies have reported that MaR1 modulates the Akt pathway in models of hepatic I/R injury, which is consistent with our findings (Tang et al., 2021). Although the specific role of Akt modulation by MaR1 has not been extensively characterized in liver fibrosis, these observations suggest a potential protective mechanism that may also contribute to fibrosis resolution. Finally, Cdk2 expression decreased after MaR1 administration, indicating that MaR1 may influence cell cycle regulation. Rather than promoting uncontrolled proliferation, the downregulation of CDK2 may reflect a reduction in aberrant cell cycle activation associated with fibrogenic cell population, thereby favouring a controlled and reparative regenerative response. Collectively, these signalling changes support a shift from stress-activated and pro-apoptotic pathways toward a regenerative and pro-resolving hepatic environment, supporting the concept that MaR1 promotes fibrosis resolution rather than simply attenuating liver injury. This interpretation is consistent with previous results indicating that MaR1 attenuates key fibrotic parameters and promotes liver regeneration (Rodríguez et al., 2021).

Liver fibrosis develops associated with chronic and sustained inflammation, which in turn leads to the activation of HSCs, mediated by immune cells, particularly macrophages. They control the progression or cessation of fibrosis based on their functional plasticity (Wang et al., 2022). While M1 macrophages exacerbate liver injury and fibrosis, M2 polarization has been associated with the resolution of fibrosis, ECM remodelling, and the restoration of liver homeostasis (Wang et al., 2023). Nevertheless, this classification hinders a comprehensive understanding of the diverse macrophage phenotypes, especially in an organ with such distinctive architecture and functions as the liver (Gao C. C. et al., 2022; Wang et al., 2021). In fact, several studies have suggested a broader and more dynamic functional spectrum of intermediate states between M1 and M2, which are induced depending on the balance of inflammatory, metabolic, and tissue stimuli (Strizova et al., 2023). Two main populations of macrophages are found in the liver under these conditions, KCs and MoMas. KCs located in hepatic sinusoids are involved in basal immune surveillance and are crucial as detectors of initial injury, as well as initiators of an inflammatory cascade within the liver (Nguyen-Lefebvre and Horuzsko, 2015). In contrast, MoMas are recruited from the bone marrow (BM) in response to chemoattractants and, once in the parenchyma, can engage in proinflammatory or reparative functions, depending on the microenvironment (Wynn et al., 2013)^.^ Although repolarization of activated macrophages towards a resolutive profile is an attractive therapeutic option, it should be noted that this reprogramming must be finely tuned, as an exaggerated shift to the M2 phenotype could increase immunosuppression and ultimately promote the progression of advanced tumours (Gao J. et al., 2022). Our findings present evidence on this type of macrophage reprogramming, as indicated by the increased number of CD206^+^ cells and the upregulation of CD206 mRNA, a widely accepted marker of the M2 phenotype (Beljaars et al., 2014). Importantly, CD206 expression not only reflects a phenotypic shift from a proinflammatory to a resolving state, but is also functionally associated with antifibrotic processes, including clearance of cellular debris, induction of ECM degrading enzymes, and to the restoration of tissue the microenvironment homeostasis (Yunna et al., 2020). This profile is consistent with a reduction in proinflammatory cytokine secretion and the activation of pro-resolving pathways. Significantly, this macrophage reprogramming occurred in parallel with fibrosis regression. In this way, previously it was reportted that MaR1 increases the population of CD206^+^ macrophages during reparative processes such as dermal wound healing and alveolar bone regeneration (Wang et al., 2020). Similarly, MaR1 has been shown to induced CD206 expression while suppressing CD80 (M1 marker) promoting a shift towards a reparative macrophage phenotype (Dalli et al., 2013). DHA has also been reported to induce M2 polarization in macrophages; for example, *in vitro* studies using MoMas showed that DHA treatment increased the expression of CD206, Arg1 and IL-10 (Kawano et al., 2019). As a therapeutic strategy, MaR1 shows distinct advantages: unlike DHA, which requires incorporation and enzymatic conversion, MaR1 acts directly as a SPM with greater potency and specificity in driving M2 polarization. In LPS-induced murine inflammation, MaR1 more efficiently upregulated CD206 and reparative mediators, highlighting its superior capacity to resolve inflammation and promote tissue repair (De Boer et al., 2014). This difference may be explained by the low and variable efficiency of endogenous DHA conversion to MaR1, whereas direct MaR1 administration ensures immediate bioavailability of the active mediator (Freedman et al., 2020).

Resident KCs play a significant role in sensing liver injury and by secreting cytokines and chemokines that promote the recruitment of MoMas. In MAFLD and other chronic liver disease, this process favours the persistence of proinflammatory macrophage populations, contributing to disease progression (Tosello-Trampont et al., 2012). Similarly, it has been documented in other liver pathologies, such as alcoholism and fibrosis, that both KCs and MoMas sustain a chronic low-grade inflammatory state associated with persistent tissue damage (Kolios et al., 2006; Ran et al., 2024). In our model, MaR1 induced KCs and MoMas toward a less inflammatory phenotype, as evidenced by increased CD206 expression, reduced CD11c levels, and a relative expansion of M0 macrophages. This finding is particularly relevant because M0 macrophages represent a non-polarized and highly plastic state, capable of differentiating in response to microenvironmental cues towards either inflammatory or reparative phenotypes (Kim et al., 2019). Previous studies suggest that maintenance of an expanded M0 macrophage pool may function as an immunological reservoir, facilitating subsequent differentiation toward an M2 phenotype and contributing to fibrosis resolution through IL-10 secretion and ECM degradation mediated by MMP1 (Zhang et al., 2022). Consistent with this concept, our findings indicate that MaR1 induced a sustained pro-resolving macrophage profile that persists for at least 5 weeks of treatment. Although a marked accumulation of fully differentiated M2 macrophages was not observed at the end of the experimental period, this may reflect an early polarization event followed by stabilization of macrophages in a plastic, functionally competent state, sufficient to maintain resolution without persistent activation (Qiao et al., 2020). Similar sustained M2-associated responses have been described in models of cerebral ischemia and colitis, where prolonged increases in CD206 expression correlated with long-term tissue protection (Zahoor et al., 2025). At the molecular level, these effects may involve nuclear receptors such as RORα, which has been strongly associated with M2 polarization and anti-inflammatory signalling, as well as LGR6, recently identified as a receptor for MaR1 in multiple tissues (Im, 2020). In line with this, Mar1 alone significantly increased hepatic M2 macrophage markers, suggesting that this mediator not only resolves ongoing inflammation but also primes the immune system toward a protective baseline state.

RORα activation has been shown to promote macrophages M2 polarization, through induction of IL-10, CD206 and Arg1, both *in vitro* and *in vivo* (Nejati Moharrami et al., 2018; Xiao et al., 2016). In diet-induced liver inflammation models, pharmacological or antibody-mediated activation of RORα increased hepatic M2/M1 ratios, reduced oxidative stress and normalized metabolic parameters (Han et al., 2017; Han et al., 2019). Accordingly, it is reasonable to propose that MaR1 exerts part of its immunomodulatory and metabolic effects through RORα activation (Jetten, 2009). RORα also induce peroxisome proliferator-activated receptor gamma coactivator-1 alpha (PGC-1α) expression, a master regulator of mitochondrial biogenesis and metabolic homeostasis, thereby amplifying anti-inflammatory transcriptional programs (Cheng et al., 2024). This MaR1/RORα/PGC-1α axis may underlie the sustained immunometabolic reprogramming observed in our model. Consistently, reduced RORα expression has been linked to accelerated fibrosis progression in MAFLD and MASH, while its reactivation confers hepatoprotection (Nematisouldaragh et al., 2024). Although MaR1-mediated RORα activation has been reported in metabolic liver disease models (Han et al., 2019), the mechanisms by which this axis operates in advanced liver disease remain poorly characterized (Nematisouldaragh et al., 2024). Our data therefore expands the relevance of this pathway to advanced fibrotic settings, suggesting that MaR1 may exert durable therapeutic effects by coordinating inflammatory resolution, metabolic restoration and tissue protection.

Interestingly, while RORα is classically described as a monomeric nuclear receptor (Im, 2020), our findings suggest a potential interaction between RORα and the p65 subunit of NF-κB under chronic inflammation conditions. Given that p65 contains the transcriptional activation domains required for NF-κB driven gene expression, this interaction may represent a functionally relevant mechanism for modulating proinflammatory transcription (Nematisouldaragh et al., 2024). Similar transrepressive mechanisms have been described for other lipid activated nuclear receptors, such as PPARα and LXR (Yoshikawa et al., 2003). Moreover, RORα has been reported to stabilize IκBα and interfere with protein kinase complex (IKK) complexes activity, thereby limiting NF-κB nuclear translocation and transcriptional output (Delerive et al., 2001; Kim et al., 2023). Competition for transcriptional coactivators, such as p300, may further contribute to this regulatory effect (Kane and Means, 2000). Collectively, these mechanisms support a role for RORα as a central regulator of macrophage function and inflammatory resolution in chronic liver disease.

Altogether, these findings identify MaR1 as a pharmacological activator of a RORα-centred pro-resolving network that links macrophage reprogramming to the suppression of NF-κB–dependent inflammatory signalling, thereby promoting hepatic fibrosis resolution rather than mere attenuation of chronic liver injury. Although additional MaR1-responsive receptors may contribute to its biological effects, our data support RORα as a key molecular mediator underlying the antifibrotic and pro-resolving actions of MaR1. Collectively, these results highlight MaR1 as a natural pro-resolving mediator with clear pharmacological relevance, supporting its further evaluation as a therapeutic strategy for macrophage driven fibrosis in chronic liver disease.

## 5 Conclusion

Taken together, our findings show that MaR1 exerts potent antifibrotic and pro-resolving effects in chronic liver injury by targeting macrophage reprogramming and the RORα/NF-κB signalling axis. Beyond suppressing inflammation, MaR1 promotes hepatocellular regeneration and actively restores hepatic homeostasis through coordinated immune and transcriptional mechanisms. From a pharmacological perspective, these results positions MaR1 as a promisin therapeutic candidate for chronic inflammatory and metabolic liver disease.

## Data Availability

The original contributions presented in the study are included in the article/Supplementary Material, further inquiries can be directed to the corresponding author.
