# Supplementary material for: Maresin 1 induces resolution of hepatic fibrosis through RORα-dependent reprogramming of resident macrophages
Source: Front Pharmacol. 2026 Apr 27;17:1826739. doi: 10.3389/fphar.2026.1826739 (PMC13158560; doi:10.3389/fphar.2026.1826739)
Supplement: Supplementary file 1 [file DataSheet1.docx]

**Maresin 1 induces resolution of hepatic fibrosis through RORα-dependent reprogramming of resident macrophages**

1. **Table S1.** **Anesthetic protocol used for rats and mice and distribution of animals per group**

| **Species** | **Acepromazine (mg/kg)** | **Xylazine (mg/kg)** | **Ketamine (mg/kg)** |
| --- | --- | --- | --- |
| Rat | 2.5 (Pacifor®, Drag-Pharma, Chile) | 5 (Xilagesic®, Drag-Pharma) | 75 (Ketamil®, Troy Laboratories, Australia) |
| Mouse | 1 (Pacifor®, Drag-Pharma, Chile) | 5 (Xilagesic®, Drag-Pharma) | 100 (Ketamil®, Troy Laboratories, Australia) |

Footnote: Doses were administered intraperitoneally. Each experimental group included 6 animals (n=6).

1. **Table S2. Primary antibodies used for Western Blot**

| **Antibody** | **Catalog Number** | **Dilution** | **Diluent** | **Brand** |
| --- | --- | --- | --- | --- |
| MMP-1 | sc-21731 | 1:1000 | 5% Milk | Santa Cruz Biotechnology |
| TIMP-1 | sc-21734 | 1:1000 | 5% Milk | Santa Cruz Biotechnology |
| α-SMA | sc-53142 | 1:1200 | 5% Milk | Santa Cruz Biotechnology |
| TGF-β | #3709 | 1:1250 | 5% Milk | Cell Signaling Technology |
| TGFβRII | sc-400 | 1:250 | 5% BSA | Santa Cruz Biotechnology |
| NF-κB p65 | sc-372 | 1:750 | 5% Milk | Santa Cruz Biotechnology |
| IκBα | 07-1483 | 1:1500 | 5% Milk | Merck Millipore |
| p-IκBα | sc-8404 | 1:1000 | 5% Milk | Santa Cruz Biotechnology |
| RORα | bs-1154R | 1:1000 | 5% Milk | Bioss Antibodies |
| GAPDH | sc-25778 | 1:3000 | 5% Milk | Santa Cruz Biotechnology |
| Histone H1 | sc-8030 | 1:1000 | 5% Milk | Santa Cruz Biotechnology |

Footnote: MMP-1: Matrix Metalloproteinase-1, TIMP-1: Tissue Inhibitor of Metalloproteinases-1, α-SMA: Alpha-Smooth Muscle Actin, TGF-β: Transforming Growth Factor Beta, TGFβRII: Transforming Growth Factor Beta Receptor II, NF-κB p65: Nuclear Factor kappa-light-chain-enhancer of activated B cells, p65 subunit, IκBα: Inhibitor of kappa B alpha, p-IκBα: Phosphorylated Inhibitor of kappa B alpha, RORα: Retinoic acid-related Orphan Receptor Alpha, GAPDH: Glyceraldehyde 3-phosphate Dehydrogenase, Histone H1: Histone H1

1. **Table S3. Sequences of specific primers used in this study.**

| **Gen** | **Secuencia Forward (5'→3')** | **Secuencia Reverse (5'→3')** | **Especie** | **NCBI** |
| --- | --- | --- | --- | --- |
| ERK1 | CTTATCAACACCACCTGCGA | TGGTGTAGCCCTTGGAGTTA | Mus musculus | NM_011952 |
| Cdk2 | GCAGAAACTCTGGATGGACC | ACAGTGAAAGGACGTTGGAC | Mus musculus | NM_009870 |
| JNK | TAAGAATGGTGCTGCTCCTG | GTTCATTCCATGAAGCCCCT | Mus musculus | NM_016700 |
| AKT | CCTCAGGGTGAGGGATACAG | CGGGCACATACTCATCAGAC | Mus musculus | NM_009652 |
| Bmal1 | CCCTAGGCCTTCATTGGATTT | GCAAAGGGCCACTGTAGTT | Mus musculus | NM_007489 |
| RORα | TTTCAGGAGAAGTCAGCAGAG | CTGCTGGTCCGATCAATCAA | Mus musculus | NM_013646 |
| TNFα | CCCTCACACTCAGATCATCTTCT | GCTACGACGTGGGCTACAG | Mus musculus | NM_013693 |
| IL-6 | TAGTCCTTCCTACCCCAATTTCC | TTGGTCCTTAGCCACTCCTTC | Mus musculus | NM_031168 |
| IL-10 | TTACCTGGTAGAAAGTGATGC | CCTTTGTCTTGGAGCTTATT | Mus musculus | NM_010548 |
| CD86 | CTTACGGAAGCACCCACGAT | CGGCAGATATGCAGTCCCAT | Mus musculus | NM_019388 |
| CD206 (Mrc1) | AGACGAAATCCCTGCTACTG | CACCCATTCGAAGGCATTC | Mus musculus | NM_008625 |
| NF-κB (p65, Rela) | CAAAGACAAAGAGGAAGTGCAA | GATGGAATGTAATCCCACCGTA | Mus musculus | NM_009045 |
| HPRT1 | GATGGAATGTAATCCCACCGTA | AGCCTAAGATGAGCGCACTGAA | Mus musculus | NM_013556 |
| GAPDH | TGTGACTTCAACAGCAACTC | TGGTCCAGGGTTTCTTACTC | Mus musculus | NM_008084 |

ERK-1: Extracellular signal-regulated kinase 1; Cdk2: Cyclin-Dependent Kinase 2; JNK: c-Jun N-terminal kinase; AKT: Protein kinase B; Bmal1: Brain and muscle ARNT-like protein 1; RORα: Retinoic acid receptor-related orphan receptor Alpha; TNFα: Tumor necrosis factor Alpha; IL-6: Interleukin 6; IL-10: Interleukin 10; CD86: Cluster of differentiation 86; CD206: Cluster of differentiation 206 / Mannose receptor C-type 1; NF-κB: Nuclear factor kappa-light-chain-enhancer of activated B cells; HPRT1: Hypoxanthine-guanine phosphoribosyltransferase 1; GAPDH: Glyceraldehyde-3-phosphate dehydrogenase

1. **Table S4. Main pharmacokinetic parameters**

| **Parameter** | **Unit** | **Value** |
| --- | --- | --- |
| t1/2 | min | 433,3125891 |
| Tmax | min | 30 |
| Cmax | pg/ml | 2909,416667 |
| C0 | pg/ml | 1871,52 |
| Clast_obs/Cmax |  | 0,450863575 |
| AUC 0-t | pg/ml*min | 512742,8 |
| AUC 0-inf_obs | pg/ml*min | 1332767,471 |
| AUC 0-t/0-inf_obs |  | 0,384720374 |
| AUMC 0-inf_obs | pg/ml*min^2 | 890974976,7 |
| MRT 0-inf_obs | min | 668,514948 |
| Vz_obs | (μg)/(pg/ml) | 0,000562863 |
| Cl_obs | (μg)/(pg/ml)/min | 9,00382E-07 |
| Vss_obs | (μg)/(pg/ml) | 0,000601919 |

Footnote: t1/2: elimination half-life; Tmax: time to maximum plasma concentration; Cmax: maximum plasma concentration; C0: initial plasma concentration; Clast_obs/Cmax: ratio of last observed concentration to maximum concentration; AUC0–t: area under the concentration–time curve from time 0 to last measurable concentration; AUC0–inf_obs: area under the concentration–time curve from time 0 extrapolated to infinity; AUC0–t/0–inf_obs: ratio of AUC0–t to AUC0–inf_obs; AUMC0–inf_obs: area under the first moment curve from time 0 to infinity; MRT0–inf_obs: mean residence time; Vz_obs: volume of distribution (observed); Cl_obs: clearance (observed); Vss_obs: steady-state volume of distribution.

1.
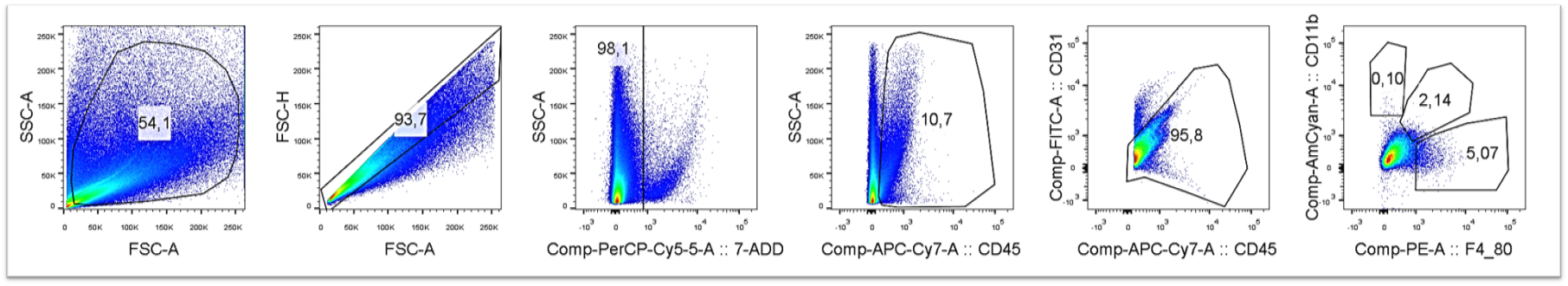
 **Figure S1. A common gating strategy for distinguishing Kupffer cells (KCs, resident hepatic macrophages) from monocyte-derived macrophages (MoMas) in mouse liver by flow cytometry typically**

Footnote:

- Panel 1: FSC-A vs SSC-A: initial cell selection and debris exclusion.
- Panel 2: FSC-A vs FSC-H: singlet selection to remove cellular aggregates.
- Panel 3: 7-AAD: exclusion of dead cells (selection of viable cells negative for 7-AAD).
- Panel 4: CD45⁺: selection of liver leukocytes.
- Panel 5: CD45⁺CD31⁻: exclusion of endothelial cells (CD31⁺).
- Panel 6: CD11b vs F4/80: identification of macrophages. KCs (F4/80^+^CD11b^low^) and MoMas (F4/80^+^CD11b^hi^) were defined within this population.

1. **Figure S2. Validation of the DEN-induced chronic liver injury model and hepatoprotective effect of MaR1.**


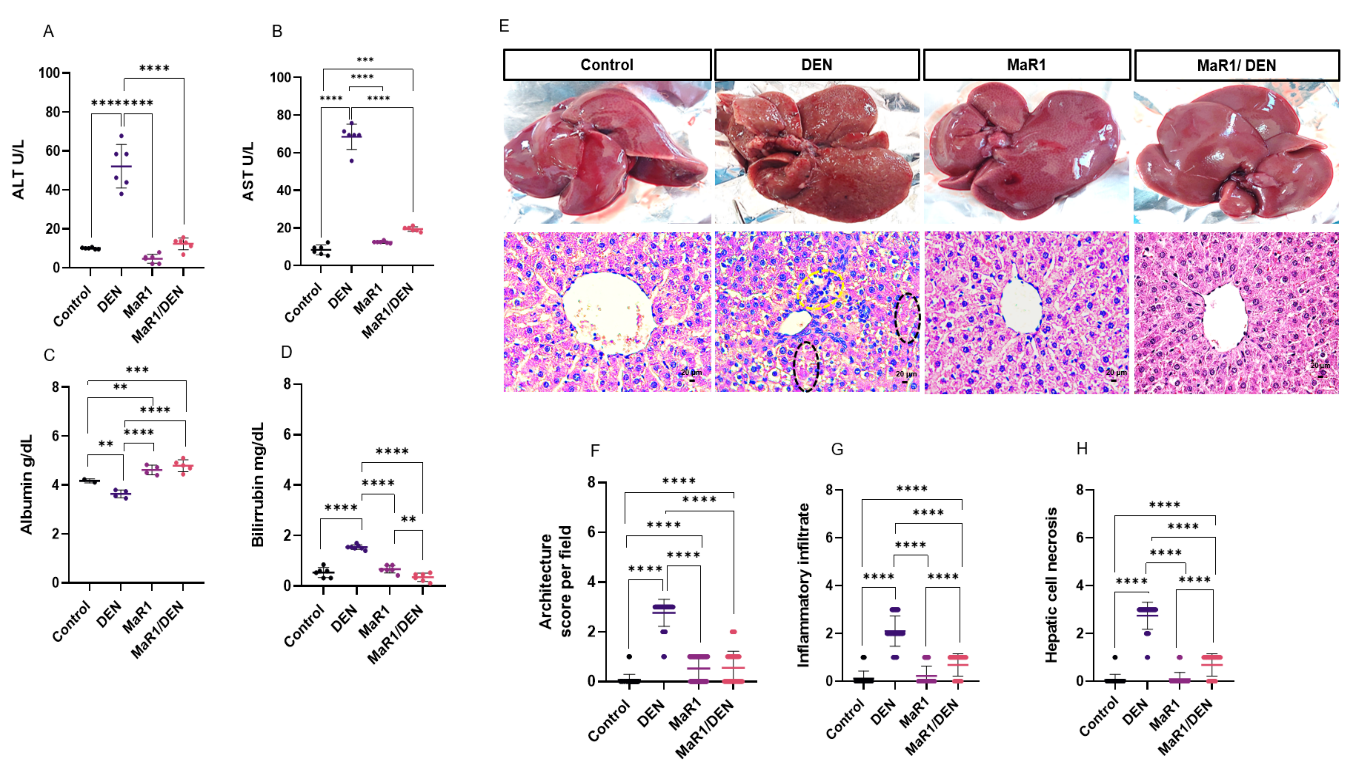


Footnote: Experimental design of DEN-induced liver injury and MaR1 treatment in SD rats. (B-E) Serum biochemical markers (ALT, AST, albumin, and bilirubin) and representative liver sections stained with H&E showing necrosis, inflammation, and architectural alterations (F). Inflammatory infiltration is indicated by yellow dashed circles, while necrotic areas are highlighted by black dashed circles. MaR1 treatment restored biochemical parameters and improved histological features. n = 6 animals per group. Scale bar = 20 µm. p < 0.05 (G-I).

1. **Figure S3. Histological validation of fibrosis and MEC deposition in the DEN model.**

**
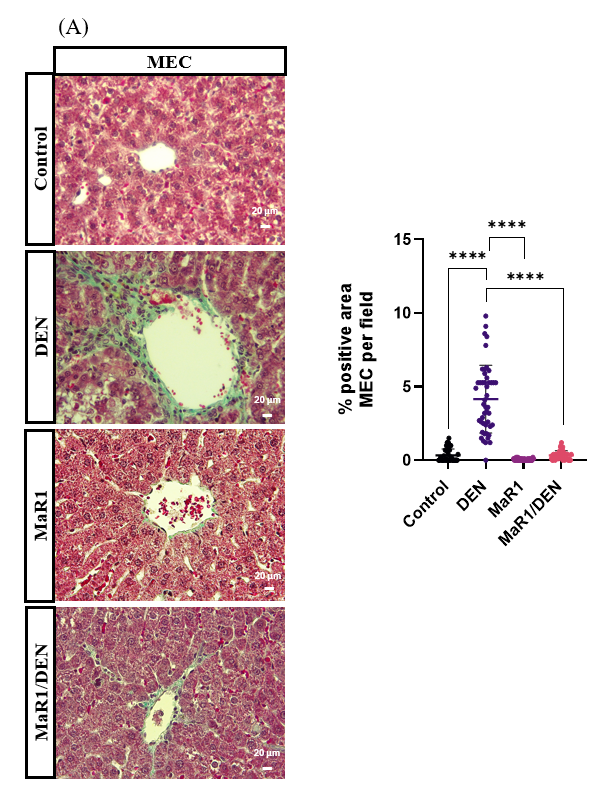
**

Footnote: (A) Representative liver sections stained with Masson’s trichrome from Control, DEN, MaR1, and MaR1/DEN groups showing fibrotic septa (blue staining). Quantification of the fibrotic area (% stained area) is shown on the right. *Scale bar = 50 µm. n = 4 animals per group. p < 0.0001.

1.
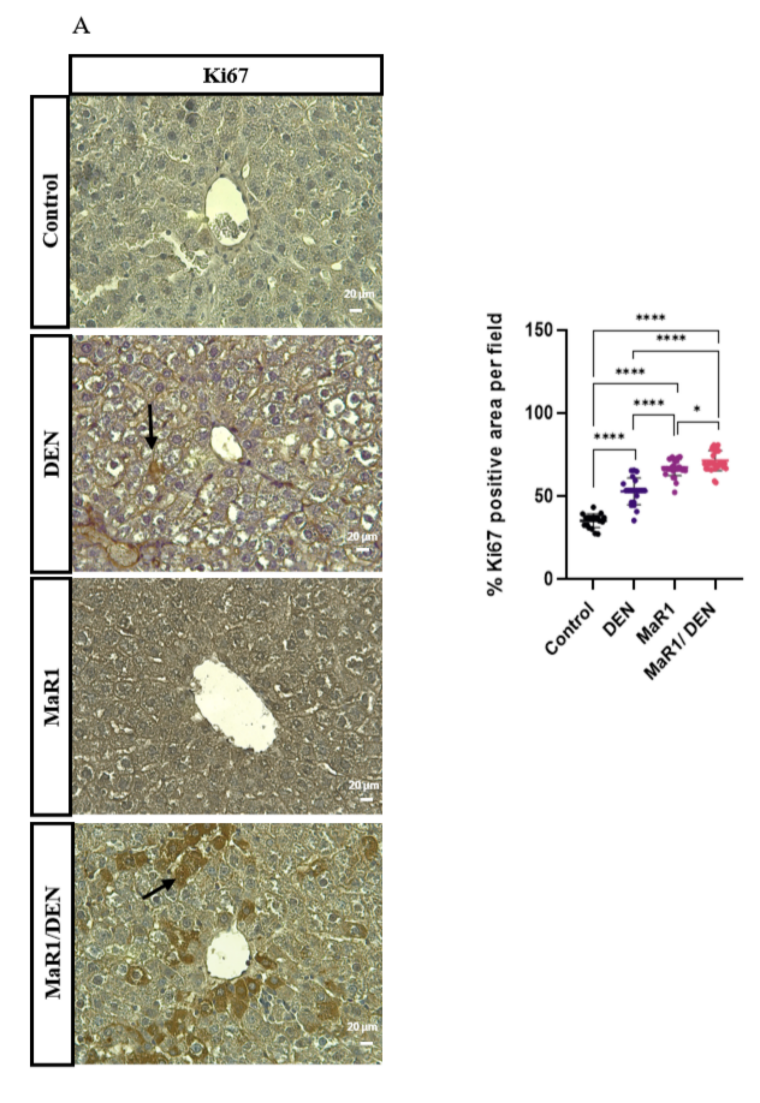
**Figure S4. MaR1 promotes hepatocellular proliferation in DEN-induced liver fibrosis.**

Footnote: (A) Representative liver sections showing Ki67 immunostaining used to evaluate hepatocyte proliferation. The MaR1-treated group showed a marked increase in Ki67-positive nuclei compared with the DEN group (p < 0.05). n = 4 animals per group. Scale bar = 20 µm.
